# Effects of Kurozu concentrated liquid on adipocyte size in rats

**DOI:** 10.1186/1476-511X-9-134

**Published:** 2010-11-23

**Authors:** Li-Tao Tong, Yoshinori Katakura, Sayaka Kawamura, Sanae Baba, Yasutake Tanaka, Miyako Udono, Yoshie Kondo, Kumi Nakamura, Katsumi Imaizumi, Masao Sato

**Affiliations:** 1 Laboratory of Nutrition Chemistry, Faculty of Agriculture, Kyushu University, 6-10-1, Hakozaki, Higashi-Ku, Fukuoka 812-8581, Japan; 2 Lavoratory of Cellular Regulation Technology, Faculty of Agriculture, Kyushu University, 6-10-1, Hakozaki, Higashi-Ku, Fukuoka 812-8581, Japan; 3 EGAO CO., LTD, 1-47, Higashihon-machi, Kumamoto 862-0902, Japan

## Abstract

**Background:**

Kurozu concentrated liquid (KCL) is used as a health-promoting supplement for the treatment of disorders such as cancer, hyperlipidemia, and hypertension in Japan. We investigated the possible anti-obesity effects of KCL in rats.

**Methods:**

Male Sprague Dawley rats were fed American Institute of Nutrition 76 formula diet and were orally administrated KCL or acetic acid at a dose of 100 mg/kg body weight or deionized water for 4 weeks. Adipocyte size, DNA content in subcutaneous adipose tissue, lipid levels in the serum and liver, and the rate of fatty acid excretion were determined. Effects of KCL on pancreatic lipase activity and 3T3-L1 preadipocyte differentiation were investigated *in vitro*.

**Results:**

In the KCL group, the average adipocyte size in subcutaneous and perirenal adipose tissues was significantly reduced. The KCL-administered rats displayed greater numbers of small adipocytes in the subcutaneous, perirenal and mesenteric adipose tissues than did rats from the other groups. In the KCL group, the DNA content in subcutaneous adipose tissue was significantly increased. The rate of fatty acid excretion was significantly increased in the KCL group. Furthermore, KCL significantly inhibited pancreatic lipase activity *in vitro*, and also significantly inhibited fat accumulation and mRNA expression of fatty acid binding protein 2 (aP2) and peroxisome proliferator-activated γ (PPARγ) in 3T3-L1 preadipocyte. The levels of serum and liver lipids, the concentration of serum glucose, and the levels of adiponectin were similar among the 3 groups.

**Conclusion:**

Oral administration of KCL decreases the adipocyte size *via *inhibition of dietary fat absorption and reductions of PPARγ and aP2 mRNA expression levels in adipocytes.

## Introduction

Obesity is a medical condition in which excess body fat has accumulated to the extent that it has an adverse effect on health. Obesity, which has an increasing prevalence worldwide, is generally recognized as a leading important cause of metabolic syndrome and is one of the most serious public health problems in developed countries [1]. In the development of obesity, the expansion of adipose tissue is initially characterized by an increase in either the number or size of fat cells [2,3]. Adipocytes are endocrine cells that can be controlled by the regulation of glucose metabolism, food intake, and energy expenditure [4]. Moreover, the size of adipocytes is a major modulator of endocrine function. For example, hypertrophic adipocytes secrete greater amounts of fatty acids and tumor necrosis factor α than do normal adipocytes. This excess secretion has been hypothesized to cause insulin resistance [5]. Thus, reduction in adipocyte size plays a key role in preventing obesity and metabolic syndrome. Recently, an increased number of people have become interested in the effects of traditional foods on the inhibition of obesity; thus, the present study focused on this function of Kurozu.

Kurozu, which has been used as a health food for a long time, is brewing rice vinegar that is produced from unpolished rice with rice bran through static-surface acetic acid fermentation at the Kagoshima prefecture in Japan. It is called to as black vinegar (Kurozu in Japanese), due to its amber color. Kurozu and rice vinegars are produced with different raw materials (Kurozu from unpolished rice with rice bran, rice vinegar from polished rice), in addition, brewing process of Koruzu is very distinctive compared with rice vinegars [6]. The entire brewing process (saccharification of raw materials, alcohol fermentation, and acetic acid fermentation) proceeds spontaneously within the same pot on natural environment of outdoor for over 1 year. Thus, kurozu contains more amino acids, vitamins, organic acids and proteins than rice vinegar [6-8]. Kurozu has been reported to inhibit tumor growth and nitrotyrosine production, promote the activity of matrix metalloproteinase (MMP)-2 and MMP-9, and have orexigenic and bactericidal functions [9]. It has been shown to prevent hypertension, improve blood fluidity, and inhibit oxidative action [8]. It has also been reported that the ethyl acetate extract of Kurozu can prevent skin carcinogenesis in mice [10] and azoxymethane-treated rats [11]. Many other biological actions of Kurozu have been reported, such as liver-specific actions, pharmacological actions on lipid metabolism, and hemorheological actions [12]. However, there is no information about the effect of Kurozu on the reduction of obesity in rats.

In the present study, we investigated the effect of Kurozu concentrated liquid (KCL) on adiposity variables in Sprague Dawley (SD) rats. Fecal fatty acid excretion, pancreatic lipase activity, fatty acid binding protein 2 (aP2) and peroxisome proliferator-activated γ (PPARγ) mRNA expression during 3T3-L1 preadipocyte differentiation *in vitro *were determined to clarify the underlying mechanism of KCL action on adiposity variables.

## Materials and methods

### Materials

KCL was provided by EGAO Co., Ltd. (Japan). The chemical composition of KCL was determined by the official method of analysis of Association of Official Agricultural Chemists (AOAC). KCL contained 56.7% moisture (AOAC 935.29), 0.9% fat (AOAC 945.16), 2.6% ash (AOAC 942.05), and 29.8% carbohydrate (Value of difference between total amount and other all compositions). Total nitrogen content in KCL was 1.63%, it determined by Kjeldahl method (AOAC 984.13). Protein, peptide and amino acid contents in KCL were 10.2%, it would estimate by the total nitrogen content multiplying conversion factor using 6.25.

### Diets and animals

Experimental diets were prepared according to the American Institute of Nutrition (AIN) 76 formula [13] with modifications. It contained 100 g/kg soy bean oil, 200 g/kg casein, 150 g/kg corn starch, 50 g/kg cellulose, 3 g/kg DL-methionine, 2 g/kg choline bitartrate, 35 g/kg mineral mixture, 10 g/kg vitamin mixture, and 450 g/kg sucrose.

Four-week-old male SD rats (n = 21) were obtained from Kyudo Co., Ltd. (Kurume, Japan). These animals were housed individually in stainless steel cages in an air-conditioned room (temperature: 21-24°C, with lights on 08:00-20:00 hours). Before the experiment, all the rats were acclimatized to the laboratory conditions for 3 days, and they were then divided into 3 groups (control, KCL, and acetic acid) so that the average body weights were same for all of these groups. The rats had free access to deionized water. The diet was given to the rats from 16:00 to 9:00 every day with pair-feeding. We orally administered 1 mL samples at 10:00 each day. KCL and acetic acid dissolved in deionized water were given to the rats at a dose of 100 mg/kg body weight, and deionized water was orally administered to the control rats for 4 weeks.

Rat feces were collected for 3 days before the rats were sacrificed. The rats were fasted for 5 h and then sacrificed by the removal of blood from the abdominal aorta under diethyl ether anesthesia. The liver, blood, right and left leg muscle, white adipose tissues (mesenteric, perirenal, retroperitoneal, and epididymal), and brown adipose tissues were excised and weighed. The livers were kept at -20°C until analysis. The blood was kept at room temperature for 30 min and then centrifuged at 1000 × g for 10 min to collect the serum. The supernatant was kept for analysis.

This experiment was carried out according to the Guidelines for Animal Experiments of the Faculty of Agriculture and the Graduate Course, Kyushu University, Fukuoka, Japan, and Law No. 105 and Notification No.6 of the Government of Japan.

### Glucose tolerance test

During the 3 days before sacrifice, the rats were deprived of food for 8 h before oral loading with glucose (6 g/kg body weight) for a glucose tolerance test. Their blood glucose levels were measured by use of ACCU-CHEK Compact Plus (Roche diagnostics Co., Ltd, Tokyo, Japan) at 0, 15, 30, 60, 90, and 120 min after oral loading. The blood glucose area under the plasma concentration time curve (blood glucose AUC) was calculated from the blood glucose levels and their time of determination.

### Analysis of morphometric and metabolic parameters

Adipocyte cell size was measured as described elsewhere [14,2]. Briefly, white adipose tissue and brown adipose tissue were rinsed with saline solution, fixed in 10% neutral formalin buffered solution, and embedded in paraffin. The tissues were then cut into 10-μm sections and stained with hematoxylin to measure cell size (100 cells/a rat) by National Institutes of Health (NIH) *Image *as measuring software. The DNA content of subcutaneous adipose tissue was extracted using proteinase K followed by phenol/chloroform. The quantity of the obtained DNA was assessed by UV spectrophotometry at 260 nm in described everywhere.

Serum total cholesterol and high-density lipoprotein (HDL)-cholesterol levels were measured using enzyme assay kits from Kainosu Co., Ltd (Tokyo, Japan). Serum triacylglycerol and phospholipid levels were measured using enzyme assay kits from Wako Pure Chemicals (Triglyceride E test and Phospholipids C test; Osaka, Japan). Serum adiponectin concentration was measured using an enzyme-linked immunosorbent assay (ELISA) kit (Mouse/rat adiponectin ELISA kit; Otsuka Pharmaceutical, Tokyo, Japan). Liver lipids were extracted by the method of Folch et al [15]. The levels of total cholesterol, triacylglycerol, and phospholipids were determined by the method of Sperry and Webb [16], Fletcher [17], and Wootton [18], respectively.

Feces were lyophilized and weighed. Fecal fatty acids were analyzed by the method of Ven de Kamer et al [19], modified by Jeejeebhoy et al [20].

### Pancreatic lipase activity *in vitro*

An emulsion (9 mL) containing 80 mg trioleoylglycerol, 10 mg phosphatidylcholine, and 5 mg sodium taurocholate in 0.1 mol/L N-tris (hydroxymethyl) methyl-2-aminoethane sulfonic acid (TES) buffer (pH 7.0) containing 0.1 mol/L NaCl was prepared by sonication and was kept at 37°C. A total of 100 μL of the emulsion was incubated with 50 μL porcine pancreatic lipase (5 U) (Nakarai Tesque, Kyoto, Japan) solubilized in 0.1 mol/L TES buffer containing 0.1 mol/L NaCl and various concentrations of sample (pH of KCL and acetic acid was adjusted to 7.0) solutions (100 μL) at 37°C for 30 min. The amount of released oleic acid was determined according to the method of Han et al [21].

### Cell culture and treatment

The 3T3-L1 preadipocytes (JCRB9014) were maintained in DMEM (Nissui Co., Ltd Tokyo, Japan) containing 10% calf serum and 10 mM HEPES, and 10% NaHCO_3_. The differentiation of preadipocytes was carried out in DMEM containing 10% fetal bovine serum (FBS), 0.5 mM 3-isobutyl-1-methylxanthine-IBMX (Wako Co., Ltd), 0.25 μm dexamethasone, and 10 μg/mL insulin for 72 h (phase I). After 3 days, the preadipocytes treated in phase I were transferred to DMEM containing 10% FBS for 48 h (phase II). Acetic acid (3%, used as a negative control) and KCL (3%) were added to DMEM during phase I or DMEM during phase I and II at the same time. The KCL was from the same lot used in the animal experiments.

Differentiated 3T3-L1 preadipocytes were fixed in 10% formalin at room temperature for 10 min and then stained with Oil-Red O (Sigma, St Louis, MO, USA) at 60°C for 15 min after washing with PBS. The 3T3-L1 preadipocytes were mixed with 4% Nonidet P-40/2-propanol for 15 min and examined at 492 nm. Total RNA was extracted from the 3T3-L1 preadipocytes with TRIzol Reagent (Invitrogen, Carlsbad, CA, USA). The cDNA was synthesized from 2.5 μg of total RNA. Gene expression levels were analyzed by quantitative real-time reverse transcriptase-polymerase chain reaction (RT-PCR) by using the SYBR Premix EX Taq II kit and Thermal Cycler Dice Real Time System TP800 (Takara, Shiga). The expression of aP2 (Primers: forward, 5'-AACACCGAGATTTCCTT-3' and reverse 5'-ACACATTCCACCAC-CAG-3') and PPARγ (Primers: forward, 5'-AGGCCGAGAAGGAGAAGCTGTTG-3' and reverse 5'-TGGCCACCTCTTTGCTCTGCTC-3') were analyzed. The mRNA levels were normalized using β-actin (Primers: forward, 5'-CAAAAGCCACCCCCACTCCTAAGA-3' and reverse 5'-GCCCTGGCTGCCTCAACACCTC-3').

### Statistical analysis

The data were expressed as means with standard errors and analyzed by Tukey-Kramer's multiple comparison post hoc test for the animal experiments and Student's *t *test for the cultured cell experiment. Statistical significance was defined as *P *< 0.05 or *P *< 0.01. The analysis was carried out with Excel 2002 (Microsoft, Redmond, WA, USA).

## Results

### Growth parameters, adipocyte size and DNA content in subcutaneous adipose tissue

Body and organ weights and several metabolism-related indicators and morphometric parameters were measured in the 3 groups (Table 1). Body weight, food intake, and feed efficiency were not different among the rats in the 3 groups. Adipose tissue, liver, and leg muscle weights were not different among the 3 groups.

**Table 1:** Effects of orally administered Kurozu concentrated liquid (KCL) on morphometric and metabolic variables

	Control (mean ± SE)*	KCL (mean ± SE)*	Acetic acid (mean ± SE)*
Food consumption (g/day)	21.0 ±0.4	21.2 ± 0.6	21.2 ± 0.6
Body weight gain (g)	237 ± 7	243 ± 9	240 ± 9
Liver mass (g/100 g body weight)	4.55 ± 0.14	4.60 ± 0.17	4.42 ± 0.16
Leg muscle (g/100 g body weight)^†^	1.13 ± 0.09	1.20 ± 0.07	1.20 ± 0.07
Fat mass (g/100 g body weight)			
Subcutaneous	4.01 ± 0.23	3.41 ± 0.20	3.57 ± 0.21
Mesenteric	1.16 ± 0.06	1.11 ± 0.07	1.23 ± 0.07
Perirenal	0.453 ± 0.032	0.416 ± 0.034	0.499 ± 0.056
Retroperitoneal	1.79 ± 0.11	1.82 ± 0.12	1.72 ± 0.09
Epididymal	1.45 ± 0.15	1.53 ± 0.16	1.50 ± 0.13
Brown adipose tissue	0.145 ± 0.010	0.132 ± 0.013	0.140 ± 0.009
Total mass	9.02 ± 0.32	8.42 ± 0.33	8.65 ± 0.38
Adipocyte size (μm^2^)			
Subcutaneous	2895 ± 247^b^	1856 ± 269^a^	4392 ± 492^c^
Perirenal	3869 ± 324^b^	2547 ± 330^a^	3657 ± 255^b^
Mesenteric	1468 ± 133^a, b^	1271 ± 96^a^	1805 ± 137^b^
DNA (μg/g Subcutaneous fat)	16.4 ± 2.5^e^	32.0 ± 4.4^d^	14.3 ± 2.1^e^
Serum			
Triacylglycerol (mg/dl)	253 ± 33	249 ± 32	185 ± 27
Total cholesterol (mg/dl)	83.3 ± 9.9	68.5 ± 8.8	74.1 ± 7.4
HDL cholesterol (mg/dl)	48.4 ± 4.7	45.6 ± 5.3	47.8 ± 2.7
Glucose (mg/dl)	140 ± 2	138 ± 4	144 ± 2
Adiponectin (μg/ml)	3.90 ± 0.19	4.48 ± 0.27	4.50 ± 0.37
Blood glucose AUC (mg/dl•h)	99.8 ± 6.0	104 ± 7	107 ± 13
Liver (mg/g)			
Triacylglycerol	24.7 ± 2.6	30.3 ± 5.6	23.5 ± 4.2
Phospholipids	27.8 ± 0.6	25.9 ± 1.4	28.9 ± 0.6
Cholesterol	3.53 ± 0.19	3.11 ± 0.31	3.18 ± 0.42
Fecal mass (g/day)	1.70 ± 0.05^g^	1.88 ± 0.03^f^	1.73 ± 0.05^g^
Fatty acid in feces (mg/day)	86.9 ± 6.4^g^	128 ± 7^f^	89.1 ± 7.6^g^
Fatty acid excretion rate (%/day)	3.91 ± 0.27^g^	5.67 ± 0.34^f^	3.96 ± 0.41^g^

* Means and standard errors (SE) were determined for 7 rats per group. Different superscript letters indicate significant differences at ^a,b,c^*P *< 0.05, ^f,g^*P *< 0.01, ^d,e^*P *< 0.001.

^† ^Leg muscle mass indicates the sum of quadriceps weight of the right and left legs.

HDL = high-density lipoprotein; AUC = area under the plasma concentration time curve.

Compared with the control group, the average adipocyte size in subcutaneous adipose tissues in the KCL group was significantly decreased (*P *< 0.05) (Table 1), but the size in the acetic acid group was increased (*P *< 0.05). The average size in perirenal adipocyte tissues in the KCL group was reduced significantly compared to that in the control and acetic acid groups (*P *< 0.05). Although there were no significant differences in the average size in mesenteric adipose tissues between the KCL and control groups, the average size in the acetic acid group was greater than that in the KCL group (*P *= 0.069).

**Figure 1:**
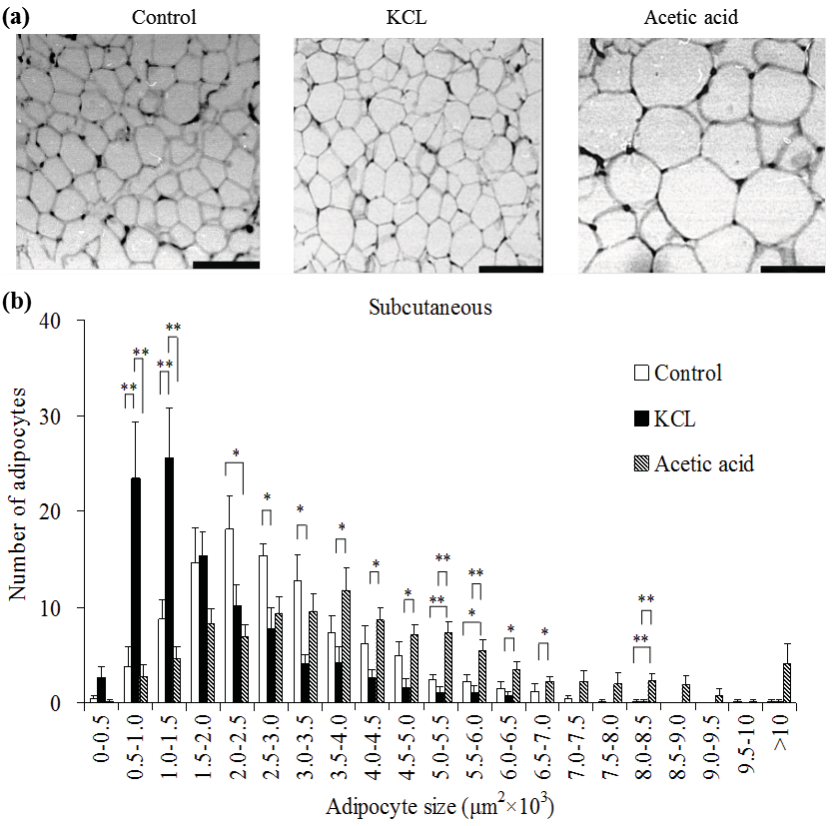
**Effect of orally administered Kurozu concentrated liquid (KCL) on cell size in white adipose tissue**. Adipocytes are shown in paraffin sections of subcutaneous (a) adipose tissue (scale bar: 100 μm). The profile of the distribution of the cell size of adipocytes from subcutaneous (b) adipose tissue. Values are means with standard errors for 7 rats per group. Different characters indicate significant differences at *P < 0.05, **P < 0.01.

**Figure 2:**
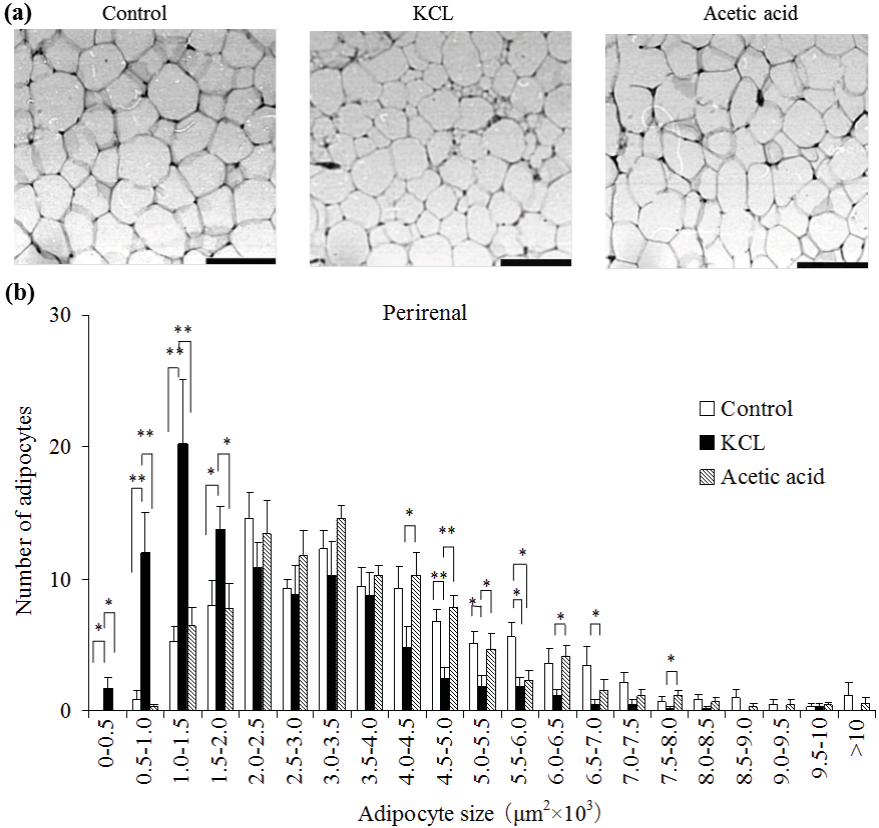
**Effect of orally administered Kurozu concentrated liquid (KCL) on cell size in white adipose tissue**. Adipocytes are shown in paraffin sections of perirenal (a) adipose tissue (scale bar: 100 μm). The profile of the distribution of the cell size of adipocytes from perirenal (b) adipose tissue. Values are means with standard errors for 7 rats per group. Different characters indicate significant differences at *P < 0.05, **P < 0.01.

**Figure 3:**
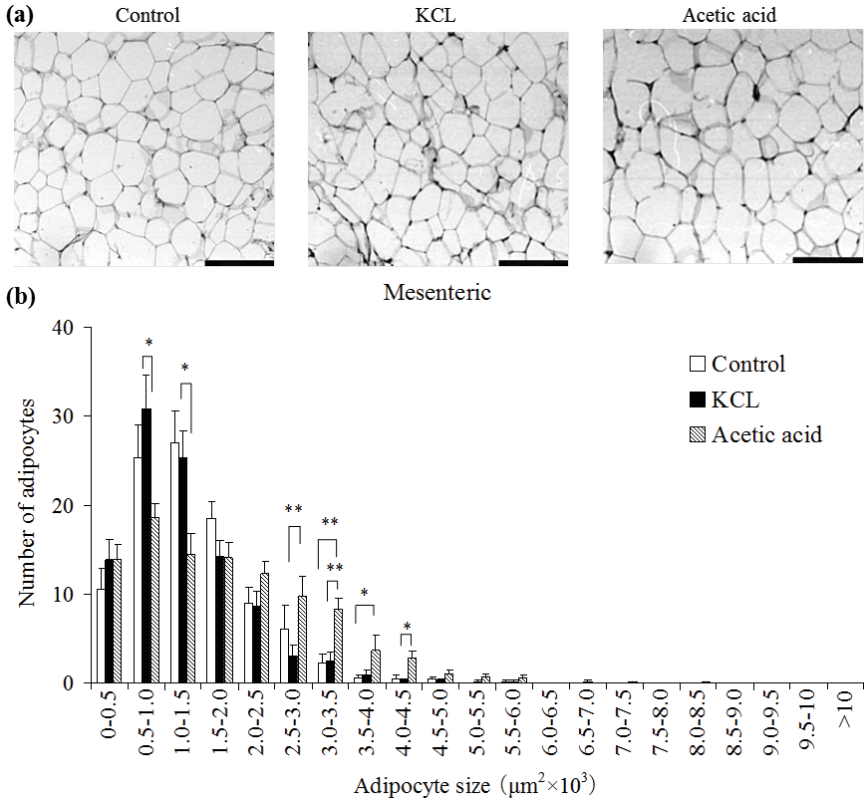
**Effect of orally administered Kurozu concentrated liquid (KCL) on cell size in white adipose tissue**. Adipocytes are shown in paraffin sections of and mesenteric (a) adipose tissue (scale bar: 100 μm). The profile of the distribution of the cell size of adipocytes from mesenteric (b) adipose tissue. Values are means with standard errors for 7 rats per group. Different characters indicate significant differences at *P < 0.05, **P < 0.01.

The number of small adipocytes was increased and the number of large adipocytes was decreased in the subcutaneous (Figure 1), perirenal (Figure 2), and mesenteric (Figure 3) adipocyte tissues in the KCL group compared with in the control and acetic acid groups. The number of large adipocytes was greater in the acetic acid group than in the control group. The DNA content, as an index of the number of cells, in the subcutaneous adipose tissue was significantly increased in the KCL group than in the control and acetic acid groups.

### Serum and liver lipid, serum glucose, and adiponectin levels

There was no obvious effect of KCL on serum triacylglycerol, total cholesterol, HDL cholesterol, glucose, blood glucose AUC, and adiponectin levels (Table 1). There were no significant differences in the liver triacylglycerol, phospholipids, and total cholesterol levels among the 3 groups.

### Feces and fatty acids

The dry weight of the feces was significantly greater in the KCL group than in the control and acetic acid groups (Table 1). Likewise, the fecal fatty acid content and the excretion rate were also significantly higher in the KCL group than in the control and acetic acid groups.

### Pancreatic lipase activity *in vitro*

**Figure 4:**
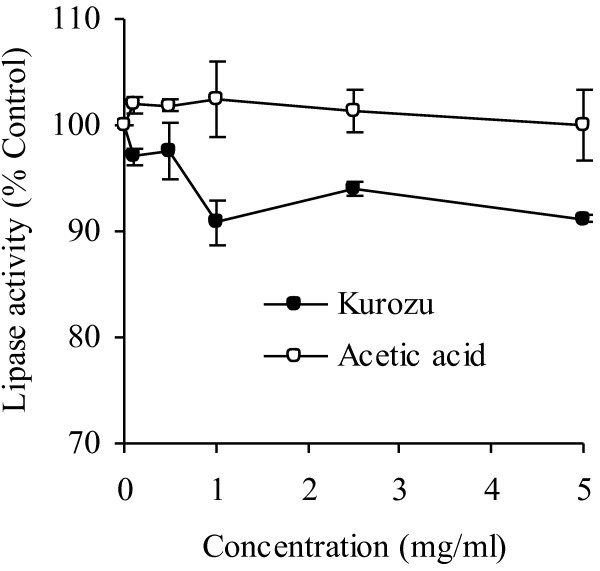
**Effect of Kurozu concentrated liquid (KCL) on the activity of pancreatic lipase *in vitro***. Values are means with standard errors for triplicate experiment.

We measured the effects of KCL and acetic acid on the activity of pancreatic lipase *in vitro *(Figure 4). Addition of KCL suppressed pancreatic lipase activity in a dose-dependent manner up to a concentration of 1 mg/mL, but this effect was not seen when acetic acid was added.

### 3T3-L1 preadipocyte differentiation and aP2 and PPARγ expression

The fat accumulation of 3T3-L1 preadipocytes in phases I and I & II was inhibited in the KCL group compared with that in the acetic acid group (Table 2). Compared with acetic acid group, the expression of aP2 and PPARγ mRNA during phases I and I & II was reduced than in the KCL group.

**Table 2:** Effect of Kurozu concentrated liquid (KCL) on 3T3-L1 preadipocyte differentiation and the expression of fatty acid binding protein 2 (aP2) and peroxisome proliferator-activated γ (PPARγ) mRNA during 3T3-L1 preadipocyte differentiation

	Phase I (mean ± SE)		Phase I & II (mean ± SE)	
		
	Acetic acid	KCL	Acetic acid	KCL
Absorbance	0.139 ± 0.002^a^	0.124 ± 0.004	0.135 ± 0.001^b^	0.125 ± 0.001
aP2/β-actin (%)	73.7 ± 0.7^b^	56.0 ± 2.8	108 ± 1^c^	32.7 ± 0.5
PPARγ/β-actin (%)	1.74 ± 0.03^c^	0.943 ± 0.032	1.36 ± 0.04	N.D.

Differentiated cells were stained with Oil Red O and their absorbance was determined at 492 nm. In the determination of mRNA levels, β-actin was used as a loading control. The means and standard errors (SE) are for triplicate experiments. Different superscript letters indicate significant differences at ^a^*P *< 0.05, ^b^*P *< 0.01, ^c^*P *< 0.001. N.D.; not detected.

## Discussion

In the present study, the average size of adipocytes was significantly smaller in the subcutaneous and perirenal adipose tissues in the KCL group than in the control and acetic acid groups, despite the fact that KCL administration did not change the adipose tissue mass. Reduction of adipocyte size is meaningful for preventing obesity because the enlargement of adipose tissue increased hypertrophy and hyperplasia can cause obesity [22]. The increase in adipocyte tissue mass in obese mice is characterized by an increase in adipocyte size up to a maximum size, followed by an increase in the number of adipocytes [23]. Hypertrophic adipocytes secrete growth factors that can trigger adipogenesis through hyperplasia, and excess secretion has been hypothesized to cause insulin resistance [5]. In the KCL group, the number of small adipocytes in the subcutaneous, perirenal, and mesenteric adipocyte tissues were increased while the number of large adipocytes was decreased. The DNA content reflects number of cells in the tissues. In the present study, DNA content in the subcutaneous adipose tissues in KCL group was significantly increased. Therefore, the adipose tissues in the KCL group possessed more small cells per a unit than the other groups. These changes in the adipocyte size distribution profile were beneficial in the prevention of obesity in SD rats and obese Zucker rats [2,3]. These results indicate that KCL plays an important role in regulating the development of obesity.

Adipocyte size in the subcutaneous abdominal depot is an important predictor for the future development of diabetes mellitus type II in humans [24]. Moreover, the oral administration of KCL has been reported to improve diabetes mellitus type II in KKA^y ^mice [25]. However, in our study, there were no differences in the serum glucose levels and blood glucose AUC levels as determined by an oral glucose tolerance test among the 3 groups. This is likely because the SD rats used in this experiment are not diabetic animal model.

Although the detailed mechanisms of the effect of KCL on the suppression of adipocyte hypertrophy are unknown, KCL effectively inhibited intestinal absorption of lipid as shown by an increased excretion of fatty acids in the feces of rates in the KCL group compared to in the control and acetic acid groups. These results show that KCL may inhibit dietary fat absorption, thus, an effect of KCL on pancreatic lipase activity is to be investigative, because pancreatic lipase is a key enzyme that promotes the hydrolysis of triglycerides from dietary fat into fatty acids and 2-monoglycerol which are then absorbed into intestinal cells [26]. It is clearly known that dietary fat is not directly absorbed from the intestine, unless it has been subjected to the action of pancreatic lipase [27]. Thereby, to improve obesity and hyperlipidemia, it may be effective to reduce fat absorption by lipase inhibition [28]. Our data showed that KCL inhibited pancreatic lipase activity in a dose-dependent manner *in vitro*. These findings raise the possibility that KCL is involved in the inhibition of dietary fat absorption through the inhibition of pancreatic lipase activity, thereby resulting in an increase in small adipocytes. It has been shown that Kurozu contains phenolic acid compounds, such as vanillic acid, sinapic acid, and ferulic acid [8]. These phenolic acids have inhibitory activity on pancreatic lipase [29].

Both aP2 and PPARγ, which are key regulatory factors in adipogenesis, are primarily found in adipose tissue. Reductions in aP2 and PPARγ mRNA expressions are involved in the suppression of hypertrophy of adipocyte [5,30,31]. In the present study, the KCL group reduced accumulation of fat and mRNA expression of aP2 and PPARγ in the 3T3-L1 preadipocyte compared with the acetic acid group. It has been reported that phenolic acids contained in KCL, such as ferulic acid, sinapic acid, and vanillic acid [8], may play a role in the control of adipogenesis by inhibiting the mRNA expression of PPARγ in 3T3-L1 cells [32]. The phenolic acids contained in KCL may inhibit hypertrophy of adipocyte in 3T3-L1 cells. Therefore, KCL may contribute to the decreased adipocyte size seen by inhibiting aP2 and PPARγ mRNA expression *in vivo*.

Acetic acid has been reported to be an effective component of metabolic regulation. In fact, dietary acetic acid has been reported to reduce serum total triacylglycerol and cholesterol levels in rats, and reduced obesity and obesity-linked type II diabetes in Otsuka Long-Evans Tokushima Fatty rats [33-35]. In a human study, acetic acid intake reduces serum total triacylglycerol and body weight in obese Japanese [36]. In the present study, the rats given acetic acid had similar levels of glucose and lipids in their serum and liver as did the control rats. The average adipocyte size in the subcutaneous adipose tissue of rats in the acetic acid group was greater than that in the control and KCL groups. In addition, acetic acid had no significant effect on pancreatic lipase activity. The obtained results clearly showed that acetic acid did not play a role in responsible for decreasing the size of adipocytes on development of obesity in SD rats fed a 10% fat diet.

## Conclusions

In summary, the results of the present study indicate that KCL decreases the adipocyte size *via *inhibition of dietary fat absorption and reductions of PPARγ and aP2 mRNA expression levels. Moreover, acetic acid as a component in KCL does not play a role in decreasing the adipocytes size on development of obesity in SD rats.

## Competing interests

The authors declare that they have no competing interests.

## Authors' contributions

LTT, YK, KN, YK, KI and MS designed the research; LTT, SK, SB, YT, MY and MS performed the research; LTT, YK and MS analyzed data; LTT, YK, KI and MS drafted the manuscript. All authors read and approved the final manuscript.
